# Influence of Dairy Manure as Inoculum Source on Anaerobic Digestion of Swine Manure

**DOI:** 10.3390/bioengineering10040432

**Published:** 2023-03-29

**Authors:** Jisoo Wi, Seunghun Lee, Heekwon Ahn

**Affiliations:** 1 National Institute of Animal Science, Rural Development of Administration, Wanju 55365, Republic of Korea; 2 Division of Animal and Dairy Science, Chungnam National University, Daejeon 34134, Republic of Korea

**Keywords:** anaerobic digestion, solid container submerged reactor, inoculation, swine manure, dairy manure

## Abstract

Inoculation is a widely used method to improve the efficiency of anaerobic digestion (AD) with a high organic load. This study was conducted to prove the potential of dairy manure as an inoculum source for AD of swine manure. Furthermore, an appropriate inoculum-to-substrate (I/S) ratio was determined to improve methane yield and reduce the required time of AD. We carried out 176 days of anaerobic digestion for five different I/S ratios (3, 1, and 0.3 on a volatile solid basis, dairy manure alone, and swine manure alone) of manure, using solid container submerged lab-scale reactors in mesophilic conditions. As a result, solid-state swine manure inoculated with dairy manure could be digested without inhibition caused by ammonia and volatile fatty acid accumulation. The highest methane yield potential was observed in I/S ratios 1 and 0.3, as 133 and 145 mL CH_4_·g^−1^-VS, respectively. The lag phase of swine manure alone was more extended, 41 to 47 days, than other treatments containing dairy manure, directly related to tardy startup. These results revealed that dairy manure can be used as an inoculum source for AD of swine manure. The proper I/S ratios leading to successful AD of swine manure were 1 and 0.3.

## 1. Introduction

The expansion of livestock industries has required proper animal manure management to minimize the negative impact on the air, water, and soil environment. Various methods have been developed, including composting, anaerobic digestion, and wastewater purification. Traditionally, most of the produced animal manure in the Republic of Korea is composted and applied to cropland. Although it has advantages in terms of nutrient recycling, it is not easy to manage odors.

Anaerobic digestion is engineered to decompose organic matter by a complex mixture of microorganisms under oxygen-free conditions [1]. Compared to composting, anaerobic digestion has been claimed to be advantageous for many reasons, including easy odor control, renewable energy (biogas) production, and small operating area requirement.

Animal manure is a suitable substrate for anaerobic digestion due to its high buffer capacity, proper C/N ratio, and abundant nutritional constituents. The demand for anaerobic digestion to stabilize animal manure and produce energy has accelerated over the past few years. Thousands of commercial biogas plants are currently being operated worldwide [2]. Most biogas plants are designed for the liquid anaerobic digestion (L-AD), which is optimized for feedstocks with solid content under 15%. However, feedstocks with high solid content, such as manure from bedded pack barns and solids separated from the solid–liquid separation process, are unsuitable for L-AD. Feedstock pretreatment methods, such as chopping and grinding, and a systematic digestion approach are applied to solve these problems.

In terms of a systematic approach, solid-state anaerobic digestion (SS-AD) is suitable for feedstocks with solid content greater than 15% [3]. Compared to L-AD, SS-AD has the advantages of a smaller reactor volume, a high organic loading rate, a low energy requirement for heating, a low parasitic loss, and a low generation of digestate [1,4,5,6]. However, low moisture content of feedstock leads retarded mass transfer, bringing low methane yield and up to three times longer retention periods in SS-AD than L-AD [1,7,8].

Agitation is one of the strategies to increase the efficiency of anaerobic digestion [9,10], but it is difficult for SS-AD due to the high solid content of the feedstock. The leachate circulation can be an alternative [11,12], but the disadvantage of energy consumption for an operation still exists. The soaking system is to submerge a solid-filled screen container in water, which can improve the efficiency of high solid feedstock AD without the energy consumption required for leachate circulation [13]. In the soaking system, the moisture content of feedstock reaches water holding capacity (WHC), which enhances volatile solid reduction and biogas production [14].

The high accumulation of intermediates of digestion, such as ammonia and volatile fatty acids (VFAs), may cause system instability and difficulty in startup [15,16,17]. In particular, solid animal manure, even though it has high potential as the feedstock of AD, presented low methane yield due to the high content of lignocellulosic particles.

Inoculation is necessary to solve the problem of solid animal manure AD [18]. The inoculum provides microbes and contributes as co-digestion feedstock, supplying nutrients and enhancing buffering capacity [19,20,21]. A suitable inoculum can increase the degradation rate, improve biogas production, shorten the starting time, and stabilize digestion [22,23]. The inocula mainly used in AD include digestate and rumen fluid [24]. The digestate can be easily gathered from anaerobic digesters, but it significantly decreases reactor utilization efficiency. It was reported that, in commercial solid animal manure anaerobic digestion plants, about 50–70% of the digestate needs to be recycled as inoculum [3,24,25]. The rumen fluid is a highly activated inoculum for lignocellulosic feedstocks [26,27], but applying it to commercial digesters is challenging because sufficient supplies cannot be secured [22].

In cattle manure, 30–70% of microorganisms are obligate anaerobes [28,29], which also contain high macro- and micronutrients that stimulate bacterial growth during anaerobic digestion. Thus, the inoculum was not required for AD of dairy manure collected from bedded pack barns [13]. Previous research also evaluated the effect of cattle manure as a co-digestion feedstock or inoculum of AD. Jain et al. [30] reported that biogas production efficiency could be improved by up to 400% when cattle manure was mixed as a co-digestion feedstock for the anaerobic digestion of food waste. Neo et al. [31] evaluated sewage sludge digestate and cattle manure as inoculum to enhance biogas production during anaerobic digestion of agricultural waste, whereby 26% more biogas was produced when using cattle manure rather than sewage sludge digestate.

A solid fraction of swine manure quickly falls into acidic inhibition in the early stage of AD due to high biodegradable volatile solid content; hence, inoculation is essential. Lee [32] suggested a proper inoculum-to-substrate (I/S) ratio of 0.5 when using sewage sludge digestate as an inoculum for a solid fraction of swine manure. Many studies have used sewage sludge digestate as an inoculation source for the anaerobic digestion of swine manure. However, it is not easy to find studies using cattle manure as an inoculum. Cattle manure as inoculum is expected to significantly improve the digester utilization efficacy, which has been pointed out as a drawback of sewage sludge digestate. Therefore, this study was conducted (1) to evaluate the availability of dairy manure as an inoculum for AD of the solid fraction of swine manure, and (2) to investigate the proper I/S ratio.

## 2. Materials and Methods

### 2.1. Physicochemical Characteristics of Inoculum and Substrate

Dairy manure (inoculum) was collected from a bedded pack dairy barn in the Animal Resources Research Center of Chungnam National University, Republic of Korea. Swine manure (substrate) was obtained from a centralized animal manure treatment plant. We used the solid fraction of swine manure collected from the decanter centrifuge animal manure solid–liquid separating system. The collected inoculum and substrate were stored at 4 °C and incubated at 37 °C for 24 h before the experiment setup. The characteristics of the inoculum and substrate are shown in Table 1.

### 2.2. Microbial Analysis of Inoculum and Substrate

The microbial composition of dairy manure (inoculum) and swine manure (substrate) was analyzed. According to the manufacturer’s instructions, DNA was extracted using a Power Food Microbial^®^ DNA Isolation Kit (MO BIO laboratories Inc., Carlsbad, CA, USA).

The extracted DNA was quantified using PicoGreen (Invitrogen, Waltham, WA, USA). From the extracted DNA, PCR was performed using a primer set targeting V3–V4 of the 16S rRNA gene for the bacteria and the ITS2 region for the archaea. Then, 2 ng of DNA was PCR-amplified with 5× reaction buffer, 1 mM of dNTP mix, 500 nM each of the universal F/R PCR primer, and Herculase Ⅱ fusion DNA polymerase (Agilent Technologies, Santa Clara, CA, USA). The cycle conditions for PCR were 3 min at 95 °C (initial denaturation), 30 s at 55 °C (annealing), and 30 s at 72 °C (extension), followed by a 5 min final extension at 72 °C. The first PCR product was purified with Agencourt AMPureXP Reagents beads (Bechman Coulter, Brea, CA, USA).

Paired-end (2 × 300 bp) sequencing was performed by Macrogen using the MiSeq 250 paired-end system (Illumina, San Diego, CA, USA). The taxonomic assignment was conducted using BLASTN (version 2.4.0.) with the NCBI 16S Microbial database [33].

### 2.3. Experimental Design and Setup

**Figure 1:**
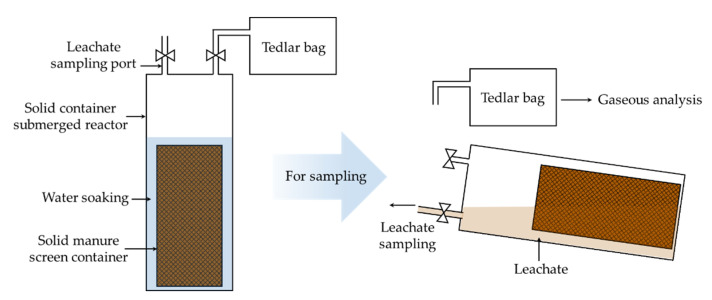
The schematic of a solid container submerged reactor used in this study.

Batch-type lab-scale anaerobic reactors (soaking system) were used in this study. The schematic of the reactor is presented in Figure 1. The plastic screen container was filled with solid animal manure and placed in each reactor. Every 2 L of the reactor was equipped with 0.7 L of the screen container. The screen container submerged the solids and separated the solid matrix from the leachate region. The swine manure was inoculated with dairy manure separately in three different ratios, 3, 1, and 0.3 (based on VS), and swine manure alone reactors were used as control (Table 2). In addition, dairy manure alone reactors were placed to estimate the contribution to CH_4_ production by inoculum. The reactor was filled with water, such that the solid manure screen container was submerged (total moisture content: 90.5%). After that, the reactors were closed with a rubber stopper, and the headspace was purged with more than three times the volume of nitrogen gas to produce anaerobic conditions. All test units were evaluated in triplicate. All 15 reactors were placed in an isothermal chamber that maintained 37 ± 1 °C for mesophilic conditions. The experimental period was 176 days. The biogas was gathered in a Tedlar bag connected to each reactor’s gas port.

During the experiment, each reactor’s gaseous and leachate characteristics were analyzed irregularly, more frequently in the early days of the experiment, to monitor rapid changes in anaerobic digestion (sampling interval: 2–9 days until day 70; after that, 2–3 weeks until the end of the experiment). The pH, VFA concentration and composition, NH_4_^+^-N concentration, and alkalinity were analyzed for the sampled leachate to monitor the stability of anaerobic digestion. On the same day, the Tedlar bag was emptied to measure the volume of biogas, and then the gaseous composition (CH_4_ and CO_2_) was analyzed.

### 2.4. Analytical Methods

The moisture content (MC) and volatile solids (VS) were determined using the standard method suggested by the American Public Health Association (APHA): weight loss after 24 h of drying at 105 °C for MC, followed by 8 h of burning at 550 °C for VS.

For sampled leachate, the pH of leachate samples was measured by a digital pH meter equipped with a combination glass electrode (Thermo Scientific, Orion 4 Star pH Conductivity Benchtop Meter). The samples were centrifuged at 3500 rpm for 30 min and filtered through 1.2 μm filter paper. Next, the volatile fatty acids (VFA), ammonium nitrogen (NH_4_^+^-N) levels, and alkalinity were determined. The VFA measurements were conducted by supernatant injection into an iGC 7200 (DS Science, Daejeon, Republic of Korea) gas chromatograph with a BP20 column (bore 0.32 mm, length 30 m, film thickness 1.0 μm) and a flame ionization detector. The photometric analysis method (Gallery Discrete Analyzer, Thermo Scientific, Waltham, MA, USA) was used to analyze NH_4_^+^-N and alkalinity in leachate.

The Tedlar bag was emptied using a graduated syringe under atmospheric conditions (25 °C, 1013 hPa) to measure the volume of the produced biogas. The analysis of methane content was performed with a gas chromatograph (iGC 7200, DS Science, Daejeon, Republic of Korea) equipped with a 1.8 m SUS column 80/100 mesh and thermal conductivity detector.

### 2.5. Estimation of Ultimate Biodegradability

Ultimate biodegradability (UB) can be determined by measuring the proportion of biodegradable volatile solids (BVSs) within the total volatile solids (TVSs). TVSs consist of BVSs and non-BVSs, but only BVSs are decomposed into biogas. Therefore, the BVS value is more suitable than the TVS value for assessing the organic matter decomposition rate. The UB of animal manure was determined using a graphical–statistical analysis [14,34], and it was used for the initial BVS mass calculation. The organic matter removal can be assumed to be the same as the mass of biogas produced due to biodegradable organic matter completely decomposed into CH_4_ and CO_2_ (Equation (1)). In this case, trace gasses contained in the biogas were excluded. The UB was calculated using a graphical method, a linear regression of the remaining volatile solid portion (TVSe) from the initial volatile solid (TVS_0_) at time *t*, as the operating time of the test approached infinity. TVS remaining at infinity was assumed to be the refractory fraction of feedstock (R_0_), which is NBVS. Then, an extrapolation of the linear plot of TVSe/TVS_0_ versus 1/time to the *y*-axis showed the refractory fraction as the value of the *y*-intercept. The remaining portion of TVSe/TVS_0_ at any time was calculated by the biogas produced during each interval. The UB of a substrate is estimated as UB = (1 − R_0_).

*BMR (biomass removed)* = *CH*_4_ *mass* + *CO*_2_ *mass*, (1)

(2) $$BMR=\frac{V_{0}\times \left(\frac{16\;g}{1\;\mathrm{mole}}\times \frac{CH_{4}}{100}+\frac{44\;g}{1\;\mathrm{mole}}\times \frac{CO_{2}}{100}\right)\;}{\frac{22.413\;l}{\mathrm{mole}}},$$

where “*BMR*” is the removed biomass (g), “*V*_0_” is the biogas volume (L) in a normal state (0 °C, 1 atm), and “*CH*_4_” and “*CO*_2_” are the contents (%) of CH_4_ and CO_2_, respectively. By substituting CO_2_ (%) = 100 − CH_4_ (%) in Equation (2), we can obtain Equation (3):

(3) $$BMR=V_{0}\times \left\{1.963-\left(0.0124\times CH_{4}\right)\right\}.$$

### 2.6. Kinetic Modeling (Modified Gompertz)

On the basis of the measured methane production, kinetic modeling was performed using the Gompertz equation to evaluate methanogenesis during AD. This estimated the cumulative methane production by using the characteristics of the methanogenic microbial growth rate. The modified Gompertz equation is presented in Equation (4) [35].

(4) $$M=P\times exp\left\{-exp\left[\frac{R_{m}\times e}{P}\left(\lambda -t\right)+1\right]\right\},$$

where “*M*” is the methane yield (L·kg^−1^-VS), “*P*” is the methane yield potential (L·kg^−1^-VS), “*R_m_*” is the maximum methane production rate (L·kg^−1^-VS·day^−1^), “*λ*” is the duration of the lag phase (days), and “*t*” is the time during digestion (days).

### 2.7. Statistical Analysis

The statistical significance of results obtained from the digesters with five different S/I ratios was evaluated using Origin Pro 8.1 software (Originlab Corporation, Northampton, UK). The significant differences in cumulative methane yield (*M*), methane production potential (*P*), maximum methane production rate (*R_m_*), lag phase (*λ*), and the time taken to achieve 95% of methane production potential (T95) were statistically analyzed using one-way ANOVA; *p*-values < 0.05 were considered statistically significant.

## 3. Results and Discussion

### 3.1. pH Changes during the Digestion

The pH value change reflects the circumstances of anaerobic digestion. The stability of the activity of acidogenic and methanogenic bacteria is directly affected by the changes in pH [36]. Ideally, the optimum pH for methanogenic bacteria is 6.8 to 7.8. Below pH 6.6 [37], the methanogenic bacterial activity is significantly reduced while acidogenic bacteria still maintain their activity, following an inhibitory effect to the anaerobic process due to VFA accumulation.

**Figure 2:**
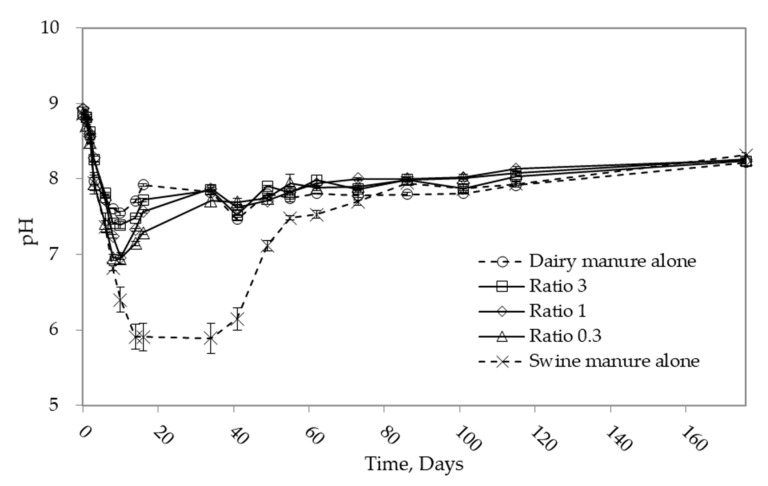
The pH changes during anaerobic digestion with different I/S ratios.

Figure 2 presents the pH changes during the anaerobic digestion of feedstocks with different I/S ratios. For treatments inoculated with dairy manure (ratios 3, 1, and 0.3) and dairy manure alone, the pH decreased and showed the lowest values on about day 10, in the range of 6.4–7.5. This is because the organic matter in the feedstock was converted to VFA through hydrolysis and acidogenesis at the beginning of anaerobic digestion. After that, the pH values recovered to 7.8–8.0 due to VFA consumption of methanogenic bacteria, and this level was maintained until the end of the experiment. However, for swine manure alone, which was not inoculated with dairy manure, the pH rapidly decreased below 5.9 and maintained an inhibitory level until day 49 of digestion due to VFA accumulation.

Neves et al. [38] reported that inoculation could overcome the acidic inhibitory effect of an anaerobic digester without pH adjustment. As a result of this study, dairy manure seems adequate for the pH stabilization of an anaerobic digester as an inoculum.

### 3.2. NH_4_^+^-N Changes during the Digestion

**Figure 3:**
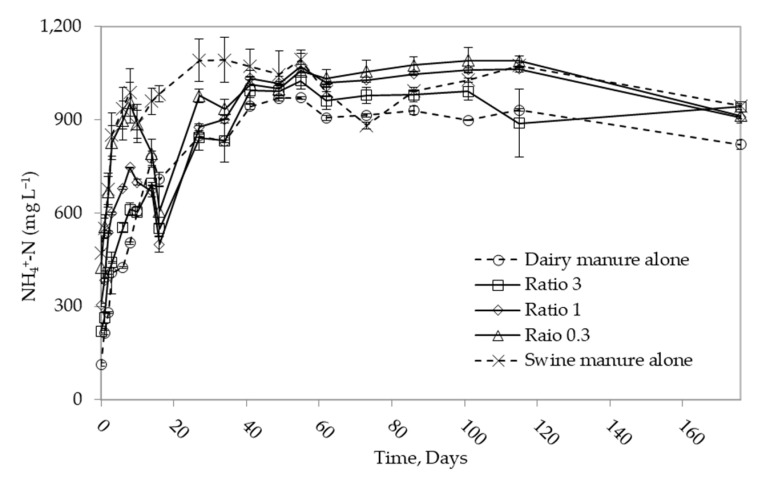
The NH_4_^+^-N concentration changes during anaerobic digestion with different I/S ratios.

The variation of NH_4_^+^-N concentration during digestion is shown in Figure 3. The NH_4_^+^-N concentration at day 0 of digestion ranged from 113 to 470 mg·L^−1^, and the values significantly increased due to the degradation and conversion of protein. The treatments with high swine manure content in the feedstock tended to show high final NH_4_^+^-N concentration: 819, 914, 941, 1064, and 1089 mg·L^−1^ for dairy manure alone, ratio 3, ratio 1, ratio 0.3, and swine manure alone, respectively. A concentration of NH_4_^+^-N greater than 1500 mg·L^−1^ inhibits the activity of methanogenic bacteria and anaerobic digestion [39]. In this research, the NH_4_^+^-N values were maintained lower than 1200 mg·L^−1^ regardless of the feedstock type during the anaerobic digestion; thus, there was no inhibitory effect of NH_4_^+^-N.

### 3.3. Total VFA and VFA/Alkalinity Ratio Changes

**Figure 4:**
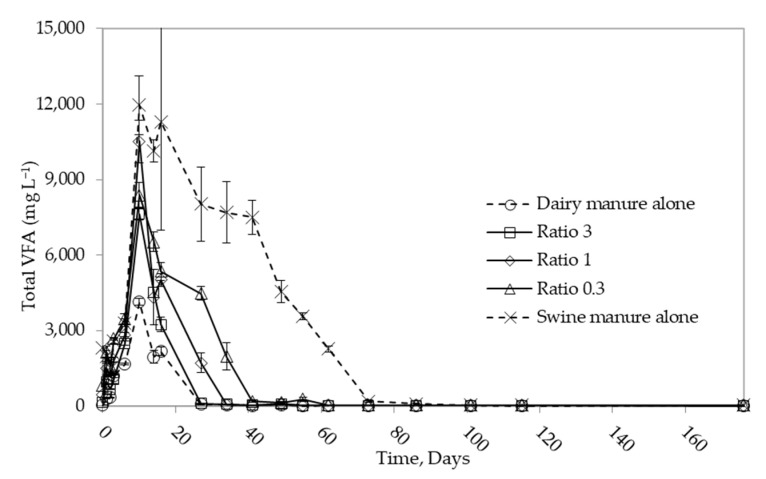
The total VFA concentration changes during anaerobic digestion with different I/S ratios.

The total volatile fatty acid (TVFA) value is one of the main parameters affecting anaerobic digestion’s stability [40]. Karthikeyan and Visvanathan [24] reported that methanogenic bacteria are inhibited due to acid accumulation when the TVFA concentration is greater than 8000 mg·L^−1^. The variation of TVFA values during anaerobic digestion is plotted in Figure 4. The maximum VFA values of each treatment peaked within day 14 and ranged from 4100 to 12,000 mg·L^−1^. Treatment with higher swine manure content required a longer time to consume VFA. For swine manure alone (not inoculated with dairy manure), the TVFA value was maintained above 8000 mg·L^−1^ for about 17 days (from days 10 to 27, Figure 4), indicating inhibited methanogenesis.

**Figure 5:**
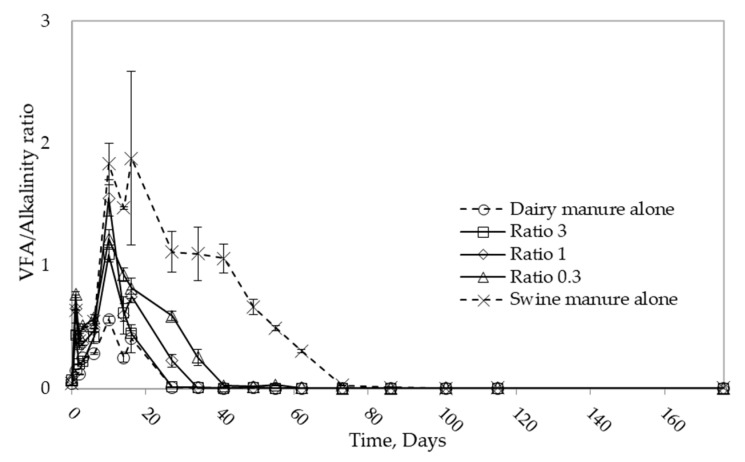
VFA/alkalinity ratio changes during anaerobic digestion with different I/S ratios.

In addition, the VFA/alkalinity ratio is presented in Figure 5. For stable anaerobic digestion, alkalinity must be maintained at the proper level. The stability of anaerobic digestion can be evaluated by the VFA/alkalinity ratio [41]. In other words, the VFA/alkalinity ratio is an indicator of the buffering capacity of the digester, and it estimates the inhibition of methanogenic bacteria that may occur according to the increased VFA. Typically, when the VFA/alkalinity ratio is less than 0.4, stable anaerobic digestion without acidic inhibition can be achieved, while a ratio greater than 0.8 leads to process failure [24]. In this experiment, the VFA/alkalinity ratio of all treatments except dairy manure alone surpassed 0.8 after day 10 of digestion. The exceeding period was more extended in treatments with higher swine manure content (Figure 5). Previous research also mentioned the buffering capacity of cattle manure when used as the co-digestion feedstock. Acosta et al. [42] reported that the co-digestion of cattle manure and cocoa waste is more stable than the mono-digestion of cocoa waste, mainly attributed to the high buffering capacity of cattle manure.

### 3.4. Ultimate Biodegradability and Volatile Solids Removal

Ultimate biodegradability (UB) was estimated by graphical statistic analysis. The UB of swine manure was 39.9% (Table 3), about 2.5 times greater than that of dairy manure (16.2%). Dairy manure is the residue after the anaerobic decomposition of feeds in the cow’s rumen. In addition, dairy manure collected from bedded pack barns contains not readily biodegradable constituents such as bedding material (sawdust, rice hull, etc.). Due to those characteristics of dairy manure, a low UB value was determined, indicating that only a small part of VS could be converted to biogas.

This study evaluated biodegradation performance based on biodegradable VS (BVS). In swine manure alone, about 67% of BVS was removed during digestion; however, when inoculated with dairy manure, the BVS removal increased to 98% (ratio 1 and ratio 0.3).

### 3.5. Methane Production and Kinetic Modeling

**Figure 6:**
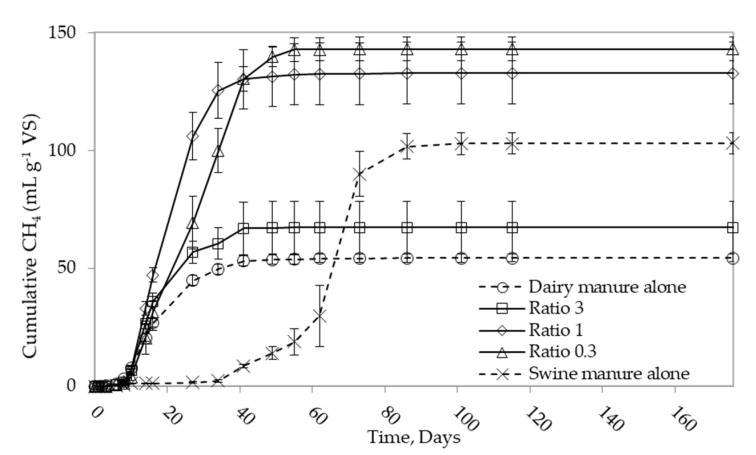
Cumulative methane production during anaerobic digestion with different I/S ratios.

Figure 6 shows the experimental cumulative methane production from treatments with different I/S ratios. Moreover, the results of modified Gompertz modeling are summarized in Table 4. The highest methane yield potential (*P*) was observed in ratio 1, ratio 0.3, and swine manure alone, at 133, 145, and 104 mL·g^−1^ VS, respectively (*p* < 0.05). This shows the same tendency as the results of previous research, whereby biogas yield was improved by adding cattle manure 30% as co-feedstock of food waste anaerobic digestion [43]. The maximum methane production rate (*R_m_*) was similar in all treatments (*p* > 0.05) except for dairy manure alone. Swine manure alone showed a long lag phase (λ) of 55 days, indicating a delayed startup of methane production of about 42–45 days compared to other treatments.

The required time (RT) for the anaerobic digestion of feedstocks depends on the feedstocks’ characteristics and the digester’s operating conditions. Determining an appropriate RT is essential for efficiently using an anaerobic digester. Korazbekova and Bakhov [44] offered that the proper RT is the time needed for cumulative methane yield to reach 95% of the feedstock’s methane potential (*P*), termed T95. In this experiment, the proper RT (T95) ranged from 31 to 36 days for dairy manure alone, ratio 3, and ratio 1 (which contained a relatively high proportion of dairy manure). Furthermore, the T95 values of ratio 0.3 and swine manure alone were 50 and 83 days, respectively (Table 4).

The methane yield potential (*P*) estimation results depended on different I/S ratios. When swine manure was digested without inoculum (in the case of swine manure alone), despite containing relatively high BVS, the VFAs cumulated rather than converted to methane, which delayed startup. However, using dairy manure as the inoculum of AD of swine manure could shorten the startup and RT and increase the methane yield.

The dairy manure used in this experiment was collected under the same conditions as the manure used for the previous study [13]. We assumed that the microbial composition of dairy manure contributed to its work as an inoculum. In the dairy manure, obligate anaerobes accounted for about 85% of total microbes, and representative microbes were *Firmicutes* (45.0%) and *Bacteroidetes* (40.4%) [13]. These organisms affected the hydrolysis of nutrients and VFA production during anaerobic digestion, further contributing to the potential of dairy manure as an inoculum source.

### 3.6. Microbial Composition of Inoculum and Substrate

**Figure 7:**
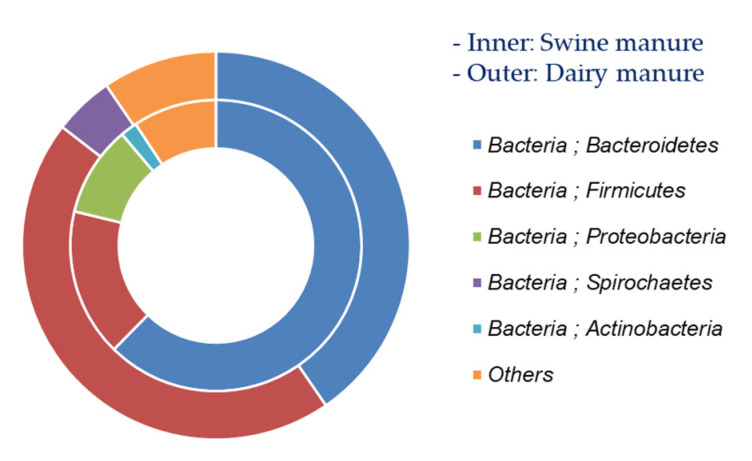
Microbial composition of dairy manure and swine manure at phylum level.

The microbial composition of dairy manure (inoculum) and swine manure (substrate) was analyzed by 16S rRNA sequencing. In dairy manure, *Firmicutes* (45.0%) and *Bacteroidetes* (40.4%) were dominant at the phylum level (Figure 7). A similar microbial composition was observed in the digestion of cattle. Mao et al. [45] reported that the microbial composition varied according to the digestive organ, and *Firmicutes* (64.8%) and *Bacteroidetes* (15.1%) were dominant overall. The swine manure microbes mainly consisted of *Bacteroidetes* (63.5%), *Firmicutes* (16.7%), and *Proteobacteria* (10.3%).

**Figure 8:**
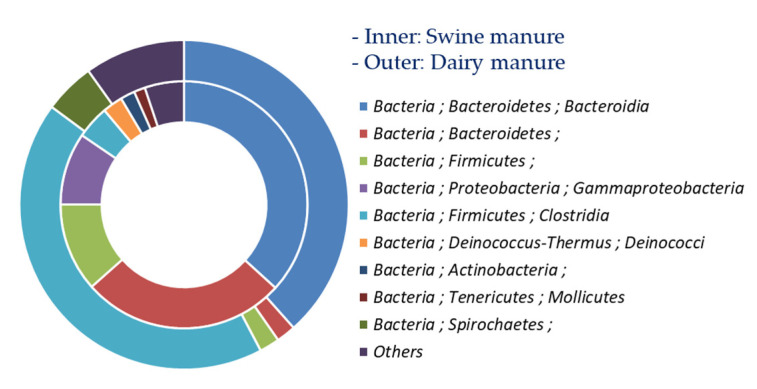
Microbial composition of dairy manure and swine manure at class level.

The *Clostridia* (class) are obligate anaerobic microorganisms, composed of microbes involved in cellulose and protein hydrolysis. *Clostridia* are commonly found in anaerobic digestion of feedstocks with high content of cellulose and protein, such as rye, chicken manure, and corn silage [46]. For dairy manure, about 95% of *Firmicutes* (45.0%) constituted *Clostridia* (42.8%), whereas, in swine manure, they accounted for only 4.34% (Figure 8). *Bacteroides* were the abundant microbe following *Clostridia* at the class level in dairy and swine manure, at 38.4% and 36.7%, respectively. The *Bacteroides* contribute to acidogenesis by converting monosaccharides, VFAs, and amino acids to short-chain fatty acids, CO_2_, and H_2_ (substrates of methanogenesis) [47].

## 4. Conclusions

In this research, we evaluated dairy manure from bedded pack barns as an inoculum source for the anaerobic digestion of a solid fraction of swine manure, as well as determined the proper inoculation ratio. Dairy manure has high potential as an inoculum because it contains many obligate anaerobes and macro- and micronutrients necessary for anaerobic digestion. The digestion of a solid fraction of swine manure inoculated with dairy manure (ratio 3, ratio 1, and ratio 0.3) operated without inhibition. In contrast, the pH value was maintained below the optimum level (6.6–7.8) until day 41 in swine manure alone due to VFA accumulation. The inoculation of dairy manure could shorten the lag phase by up to 44 days. The highest methane yield potential was observed for ratio 1 and ratio 0.3. Therefore, dairy manure can be used as an inoculum for AD of swine manure, with a proper I/S ratio of 0.3 and 1. Further research is needed on whether dairy manure from bedded pack barns can be used as an inoculum source for the anaerobic digestion of other feedstocks, such as agricultural and food waste.

## Figures and Tables

**Table 1:** Characteristics of dairy manure and swine manure.

Items	Dairy Manure ^1^	Swine Manure ^2^
Moisture content (%, w.b. ^3^)	65.4 ± 0.3	72.3 ± 0.2
Volatile solids (%, d.b. ^4^)	75.5 ± 0.3	89.0 ± 0.3
Bulk density (kg·m^−3^)	600.0 ± 3.0	485.0 ± 7.5
C (%, d.b.)	38.0 ± 1.4	43.6 ± 0.1
H (%, d.b.)	5.1 ± 0.1	6.1 ± 0.1
O (%, d.b.)	28.5 ± 0.5	38.5 ± 1.2
N (%, d.b.)	5.0 ± 0.2	4.7 ± 0.4
S (%, d.b.)	0.7 ± 0.1	0.6 ± 0.1
C/N ratio	7.6	9.3

^1^ Dairy manure from bedded pack barn; ^2^ Solid fractions of swine manure; ^3^ wet basis; ^4^ dry basis.

**Table 2:** The amount of inoculum and substrate for each treatment.

Items	Inoculum (g) ^1^	Substrate (g) ^2^	Total Weight (g)
Dairy manure alone	461.8	0	461.8
Ratio 3	321.4	113.5	434.9
Ratio 1	199.9	211.7	411.6
Ratio 0.3	93.7	297.6	391.3
Swine manure alone	0	373.3	373.3

^1^ Dairy manure from bedded pack barn; ^2^ solid fractions of swine manure.

**Table 3:** Ultimate biodegradability and volatile solid removal performance with different I/S ratios.

Items	I/S Ratio				
	Dairy Manure Alone	Ratio 3	Ratio 1	Ratio 0.3	Swine Manure Alone
Initial VS (g)	120.5 ± 0.0	111.9 ± 0.0	104.4 ± 0.0	97.7 ± 0.0	92.0 ± 0.0
BMR (g)	18.2 ± 0.3	23.2 ± 2.4	37.3 ± 1.3	38.8 ± 0.9	24.4 ± 2.3
UB (%)	16.2 ± 0.2 ^a^	21.5 ± 2.2 ^a^	36.5 ± 1.2 ^b^	40.4 ± 1.4 ^b^	39.9 ± 3.3 ^b^
VS removal (%)	15.1 ± 0.3 ^a^	20.7 ± 2.1 ^ac^	35.8 ± 1.3 ^b^	39.7 ± 0.9 ^b^	26.5 ± 2.5 ^c^
BVS removal (%)	93.4 ± 1.6 ^ab^	96.6 ± 10.0 ^ab^	98.1 ± 3.5 ^a^	98.2 ± 2.3 ^a^	66.5 ± 6.3 ^b^

^a–c^ Different superscript letters in the same row denote a significant difference (*p* < 0.05).

**Table 4:** Experimental and modified Gompertz model parameters of cumulative methane production for each treatment.

Items		I/S ratio				
	Unit	Dairy Manure Alone	Ratio 3	Ratio 1	Ratio 0.3	Swine Manure Alone
Cumulative methane yield (exp.)	N·mL·g^−1^ VS	54.3 ± 1.7 ^a^	67.2 ± 11.1 ^a^	132.7 ± 13.1 ^b^	143.0 ± 5.0 ^b^	103.0 ± 4.4 ^ab^
*P*	N·mL·g^−1^ VS	53.9 ± 1.8 ^a^	67.1 ± 11.1 ^a^	132.7 ± 13.2 ^b^	144.6 ± 5.6 ^b^	104.2 ± 5.5 ^ab^
*R_m_*	N·mL·g^−1^ VS·day^−1^	3.0 ± 0.3 ^a^	4.7 ± 0.5 ^ab^	7.5 ± 0.6 ^b^	5.8 ± 0.1 ^ab^	5.8 ± 0.9 ^ab^
λ	days	7.5 ± 0.3 ^a^	8.5 ± 0.4 ^a^	9.9 ± 0.2 ^a^	13.4 ± 2.6 ^a^	54.8 ± 4.2 ^b^
T95	days	34.1 ± 2.0 ^ab^	30.6 ± 5.5 ^a^	35.6 ± 1.6 ^ab^	49.7 ± 3.3 ^b^	83.1 ± 1.7 ^c^

^a–c^ Different superscript letters in the same row denote a significant difference (*p* < 0.05).
